# Comorbidities of diabetes and hypertension in Vietnam: current burden, trends over time, and correlated factors

**DOI:** 10.1186/s12889-023-17383-z

**Published:** 2023-12-05

**Authors:** Thi Hoang Lan Vu, Thi Tu Quyen Bui, Quoc Bao Tran, Quynh Nga Pham, Duc Truong Lai, Tu Hoang Le, Van Minh Hoang

**Affiliations:** 1 Faculty of Fundamental Sciences, Hanoi University of Public Health, No. 1A Duc Thang Ward, North Tu Liem, Ha Noi, Vietnam; 2 Division of Non-Communicable Disease Control, General Department of Preventive Medicine, Viet Nam Ministry of Health, Ha Noi, Vietnam; 3 World Health Organization Country Office for Viet Nam: Healthy Life Style and Environment, No. 304 Kim Ma Str. Ba Binh, Ha Noi, Vietnam; 4 World Health Organization Country Office for Viet Nam: NCD and Health through the Life, Course team. No. 304 Kim Ma Str. Ba Binh, Ha Noi, Vietnam; 5 Hanoi University of Public Health, No. 1A Duc Thang Ward, North Tu Liem, Ha Noi, Vietnam

**Keywords:** Hypertension, Diabetes, Comorbidity, STEPs, NCD risk factors, Clustering, Vietnam

## Abstract

**Background:**

Vietnam conducted the national Noncommunicable Disease Risk-Factor Surveillance (STEPs) surveys in the years 2010, 2015, and 2021. This study aims to use STEPs data to assess the burden of comorbidity between diabetes and hypertension, analyze trends over time, and identify factors associated with this comorbidity.

**Methods:**

The study extracted data for the population aged 25–64 years old from three STEPs surveys. Survey weight was used for all estimations of prevalence and 95% CI. Correlated factors with comorbidity were examined by a multivariate logistics model.

**Results:**

The prevalence of comorbidity in 2021 was about 3.92% among Vietnamese people aged 25–64. In the last 10 years, this prevalence has increased more than 8 times (from 0.44% to 3.92%). Sub-populations demonstrating the most significant changes included the male population, people living in urban areas, and older people. Significant factors correlated with comorbidity included demographic factors, body mass index (BMI), and clustering of 4 noncommunicable diseases (NCDs) behavioral risk factors (OR = 3.48, *p* < 0.05).

**Conclusion:**

The high comorbidity between hypertension and diabetes underscores the imperative for integrated treatment and management approaches in Vietnam. Coordinated care is essential for addressing the complex interplay between these two prevalent conditions.

## Introduction

Non-communicable diseases (NCDs) are a major global health concern, especially in low-income and middle-income countries [1]. Vietnam has experienced a rapid epidemiological transition characterized by a shift from infectious diseases to NCDs as the leading causes of morbidity and mortality. It was estimated that within the Vietnamese population, 72% of the mortality burden and 66% of the disease burden can be attributed to NCDs, particularly cardiovascular disease (CVDs), cancer, diabetes, hypertension, and chronic obstructive pulmonary disease (COPD) [2]. The most common NCDs in Vietnam include cardiovascular diseases (such as hypertension and coronary artery disease), diabetes, cancer, and chronic respiratory diseases (such as COPD) [3]. Due to rapid economic development, population aging, urbanization, and changes in dietary habits and lifestyle, the prevalence of hypertension and diabetes in Vietnam has been increasing at alarming rates [4, 5].

A previous study estimated that nationally, the prevalence of hypertension and diabetes rose nearly 2 and 6 times, respectively, among the population aged 25–64 years old in Vietnam during the last 10 years [4]. As NCDs, diabetes and hypertension have the same modifiable risk factors, including tobacco use, alcohol consumption, overweight, physical inactivity, and unhealthy diet [1]. However, previous research has defined diabetes and hypertension as “intertwined conditions” because they also share their complications. Reports indicate that individuals with type 2 diabetes mellitus (T2DM) face an approximately 2.3 times higher risk of cardiovascular disease compared to those without diabetes [6]. Furthermore, between one-third and two-thirds of mortality among patients with T2DM was due to cardiovascular disease [6, 7]. There is significant overlap in the two major complications of hypertension and diabetes (i.e., macrovascular and microvascular), possibly due to the same pathologic mechanism [8, 9]. Controlling for these comorbidities is essential to reduce the risk of complications, improve the quality of life for individuals, and alleviate the economic burden on healthcare systems. Therefore, the comorbidity of type 2 diabetes mellitus and hypertension (T2DM-HTN) has become an interesting research topic in preventing/managing NCDs [10]. Some previous studies reported T2DM-HTN in clinical settings in developed countries [11]. However, it is critical to understand the magnitude and factors associated with this comorbidity for policymaking and implementation of the intervention strategy. Vietnam conducted three rounds of the STEPs surveys [12] (i.e., a survey measuring NCDs risk factors at the population level) in the years 2010, 2015, and 2021. The survey consisted of three steps: (1) STEP 1 was for collecting demographic information/behavioral risk factors in an interviewer-administered survey; (2) STEP 2 was for collecting physical measurements such as height/weight/blood pressure; and (3) STEP 3 was for obtaining blood samples to test for glucose/cholesterol and urine samples. This study aims to combine the data from the three round surveys to explore the current burden of T2DM-HTN comorbidity, the trend over time as well as factors correlated with the comorbidity during the last 10 years among the general population aged 25–64 years old in Vietnam.

## Methodology

### Data sources and survey populations

This analysis combined data from 3 STEPs surveys in Vietnam. The sample size, sampling method, and study subjects (i.e., aged from 18 to 69 years old) were similar for the STEPs 2015 and 2021. The two recent rounds applied two stage-random systematic sampling methods (i.e., the primary sampling unit was Enumeration Areas) with a sampling frame consisting of 15% of the population of Vietnam and representing all 63 provinces and cities. In STEPs 2015, the final sample size for STEPs 1, 2, and 3 was 3,758 (response rate 97.4%), 3,036 (response rate 78.7%), and 2,816 (response rate 73.0%), respectively. In STEPs 2021, the sample size included 4,738 subjects in STEP1 (response rate of 94,76%) and 3,712 subjects in STEPs 2 and 3 (response rate of 74.2%). The 2010 survey applied a three-stage sampling method and only collected data from 8 provinces, representing 8 ecological regions of Vietnam.

The random sampling method was conducted as follows. In the first stage, utilizing the master sample frame of the General Statistics Office of Vietnam, two stratifications were created: (1 = urban; 2 = rural, and within each group divided into 3 sub-groups—coastal, lowland, and mountainous, resulting in 6 strata). In each stratum, the sampling of Primary Sampling Units (PSUs) applied the probability proportional to size (PPS) sampling method to select the required number of Enumeration Areas (EAs) in that stratum. During the second stage of sampling, households in each selected EA were chosen randomly from the sampling frame of the EAs. Subsequently, one eligible person was randomly selected from each chosen household for the STEPS 1 interview. The selection of this individual is automatically executed by the Android tablet program after eligible household members are entered into the Android tablet.

**Fig. 1:**
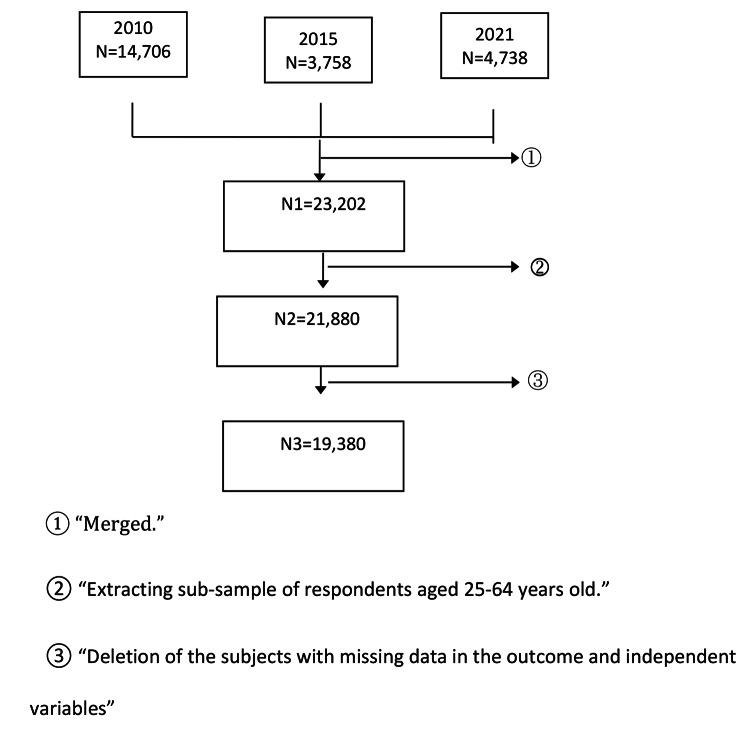
Derivation of the study sample

The study subjects in this round only include people aged 25–64 years old. The survey sample size in STEPS 2010 was 14,706 people. Panel data for 3 waves of STEPs were constructed in this study. As the subject’s age-selected criteria differed across the three STEPs, we selected data for the population aged 25–64. Figure 1 shows the derivation of study samples from datasets. After applying the exclusion criteria, the study samples comprised 19,380 observations for the analyses.

### Key measurement

#### Study outcomes

##### Hypertension

defined as an average of three measured systolic blood pressures (SBP) ≥ 140 mmHg, and/or an average of three measured diastolic blood pressure (DBP) ≥ 90 mmHg, and/or self-reported previous diagnoses of hypertension by a health professional, and/or self-reported current treatment for hypertension with antihypertensive medications in the previous 2 weeks [13].

##### Diabetes

defined as measured blood sugar (plasma venous value) ≥7mmol and/or who were currently on medication for diabetes [13].

##### Comorbidity of hypertension and diabetes

a person having both hypertension and diabetes.

#### Independent variables

##### Age group

A categorical variable with 4 classes, 25–34 years old, 35–44 years old, 45–54 years old, and 55–64 years old.

##### Geographical area

a binary variable (1 for urban and 2 for rural).

##### High body mass index (BMI)

BMI was calculated as weight (kg)/height^2^ (m). High BMI was defined as subjects with a BMI score equal to or greater than 25.

##### Clustering behavioral risk factors

A categorical variable (ranging from 0 to 4) combining 4 NCD behavioral risk factors (i.e., current smoking, current drinking, not meeting levels of physical activity recommended by The World Health Organization (WHO), and not consuming enough vegetables/fruit per day). The 4 NCD risk factors were defined as follows: (1) Current smoking: Respondents were asked the question, “Do you currently smoke tobacco on a daily basis, less than daily, or not at all?” and were defined as current smokers if the participants chose currently smoke tobacco daily or less than daily; (2) Current drinker: respondents were defined as current drinker if they consumed at least one standard drink of alcohol thin the past 30 days; (3) Not enough physical activities (PA): WHO recommendation on PA for health was “throughout a week, including activity for work, during transport and leisure time, adults should do at least an equivalent combination of moderate- and vigorous-intensity physical activity achieving at least 600 Metabolic Equivalent of Task (MET)-minutes”, so a person with total PA score in METs less than 600 MET-minutes in this study was defined as “not meeting WHO recommendation”; (4) Not eating enough fruit/vegetable: consuming less than 5 serving of fruit/vegetable per typical day.

### Statistical approach

The SVY procedure in STATA 18 was used to estimate the overall prevalence of hypertension, diabetes, comorbidity, and their 95%CI for the years 2010, 2015, and 2021. Survey weights were used for all calculations. The trends of T2DM-HTN comorbidity across subgroups of age, gender, and geographic area were also estimated. Multiple logistics regression was applied to examine correlated factors for the outcome of T2DM-HTN comorbidity. Independent variables examined in the model included survey year, age group, gender, location, BMI score, and the number of NCD behavioral risk factors. The variables for inclusion in the multivariate model were chosen using two criteria: either a p-value of the bivariate association with the outcomes < 0.2 or the variables were deemed of biological importance (e.g., gender). Two modeling strategies—enter (i.e., including all variables in the model simultaneously) and stepwise (i.e., iteratively adding or removing potential explanatory variables and testing for statistical significance after each iteration)—were assessed. Both models produced identical results; therefore, the findings from the model employing the enter method were reported. A *p*-value < 0.05 was considered statistically significant.

As the outcome focused on the comorbidity of T2DM-HTN, the inclusion of individuals with a single disease (i.e., either T2DM or HTN) in the comparison group could potentially impact the strength of the association between NCD behavioral risk factors and the outcome. Therefore, a sensitivity analysis was conducted, comparing two models:

- Model (1) utilized the entire dataset, where the outcome was comorbidity, and all individuals with only one status (either diabetes or hypertension) were placed in the group with no outcome (sample size = 19,380 subjects).
- Model (2) used data exclusively from individuals with comorbidity and those with neither hypertension nor diabetes (individuals with either disease were excluded from the analysis, sample size = 14,941 subjects).

Both models revealed the same significant predictors, with the odds ratios (ORs) in the second model slightly higher. However, given that the study objective was to identify correlated factors of comorbidity, the results of the first model were reported. We also checked the autocorrelation in a final regression model’s output with the Durbin-Watson (DW) test, the DW statistic was equal to 1.79, indicating zero autocorrelation.

### Ethical consideration

The paper was based on secondary data from the STEPS 2010, 2015, and 2021, with all identifying information removed. All procedures performed in STEPs involving human participants were in accordance with the ethical standards of The Ethical Review Board for Biomedical Research. The original STEPS surveys were approved by the Ethics Committee of the Vietnam Ministry of Health and the Tasmanian Health and Medical Human Research Ethics Committee in 2010 and the Hanoi School of Public Health in 2015 and 2020. All information on the original dataset was collected confidentially.

## Results

### The current burden of hypertension, diabetes, and their comorbidity in the year 2021

As presented in Table 1, in 2021, the prevalence of HTN among respondents aged 25–64 was 28.3% (male 36.8% and female 20.1%). This figure was higher among subjects who lived in urban than those who lived in rural (30.6% vs.27.0%). The prevalence of hypertension steadily rises with age, with the lowest prevalence in the aged 25–34 (10.7%) and the highest prevalence in the aged 55–64 (55.3%).

About 7% of respondents aged 25–64 had diabetes (male 7.9% and female 6.1%); this proportion was higher among urban respondents than rural respondents (9.0 compared to 5.8). The proportion of diabetes also increases with the age of respondents.

Among respondents aged 25–64, the co-occurrence of T2DM-HTN was observed in 4.7% of men and 3.2% of women. The prevalence of T2DM-HTN comorbidity was highest among those aged 55–64 and lowest among those aged 25–34. Approximately 13.8% of individuals diagnosed with hypertension also had diabetes, while 56.3% of individuals with diabetes also had hypertension. These prevalence rates increased with the age of the patients. Table 1, in its 5th and 6th columns, presents the estimated cases of comorbidity among 100 patients with hypertension and 100 patients with diabetes. In the oldest age group (55–64), nearly 20 out of 100 patients with hypertension also had diabetes, and approximately 73 out of 100 patients with diabetes also had hypertension.

**Table 1:** Current burden of hypertension, diabetes, and their comorbidity among the general Vietnam population in 2021

Characteristics of respondences	Prevalence of HTN [CI95%]	Prevalence of diabetes [CI95%]	Prevalence of T2DM-HTN comorbidity [CI95%]	# comorbidity among 100 HTN cases	# comorbidity among 100 diabetes
All population (N = 19,380)	28.3 [26.3, 30.3]	7.0 [5.9, 8.0]	3.9 [3.1, 4.7]	13.8	56.3
Male (n = 8,941)	36.8 [33.6, 39.8]	7.9 [6.2, 9.5]	4.7 [3.4, 6.0]	12.7	59.6
Female (n = 10,439)	20.1 [17.7, 22.3]	6.1 [4.8, 7.5]	3.2 [2.2, 4.1]	15.9	52.3
Urban (n = 6,432)	30.6 [27.1, 34.1]	9.0 [7.2, 10.8]	4.6 [3.3, 5.9]	15.2	51.4
Rural (n = 12,948)	27.0 [24.7, 29.3]	5.8 [4.6, 7.1]	3.5 [2.6, 4.5]	13.1	60.4
Age 25–34 (n = 3,979)	10.7 [7.7, 13.7]	2.3 [0.5, 4.0]	1.0 [0.2, 2.1]	9.7	45.5
Age 35–44 (n = 4,886)	23.9 [18.9, 28.9]	6.4 [3.9, 8.8]	2.1 [0.6, 3.6]	8.8	33.0
Age 45–54 (n = 5,216)	33.1 [29.0, 37.2]	6.8 [4.7, 8.8]	3.9 [2.3; 5.6]	11.9	58.0
Age 55–64 (n = 5,299)	55.3 [51.6, 58.9]	15.1 [12.4, 17.7]	11.0 [8.5, 13.4]	19.8	72.7

### The trend of hypertension, diabetes, and DM-HTN comorbidity over time

Table 2 presents the prevalence of hypertension and diabetes and their comorbidity over time. The prevalence of T2DM-HTN comorbidity was only 0.44% among the general population aged 25–64 in 2010. However, this figure dramatically rose to 3.92% in 2021 (Cochran–Armitage test for trend, *p* < 0.001). For all three outcomes, the increasing trend was more significant between the years 2021 and 2015 than between the years 2015 and year 2010. For instance, for T2DM-HTN comorbidity, the absolute difference in prevalence between 2010 and 2015 was 1.5%, while this figure was nearly 2% between 2015 and 2021.

**Table 2:** Trends of T2DM-HTN comorbidity among the population aged 25–64 in Vietnam over the last 10 years

	*Year 2010* *Prevalence [95%CI]*	*Year 2015* *Prevalence [95%CI]*	*Year 2021* *Prevalence [95%CI]*
Hypertension	15.85 [15.04, 16.65]	20.78 [18.95, 22.60]	28.33 [26.34, 30.32]
Diabetes	1.19 [0.89, 1.48]	3.87 [3.05, 4.70]	6.96 [5.89, 8.03]
Comorbidity	0.44 [0.33, 0.55]	1.93 [1.38, 2.48]	3.92 [3.14, 4.70]

### The trend of hypertension, diabetes, and comorbidities over time by gender, geographical, and age groups.

**Fig. 2:**
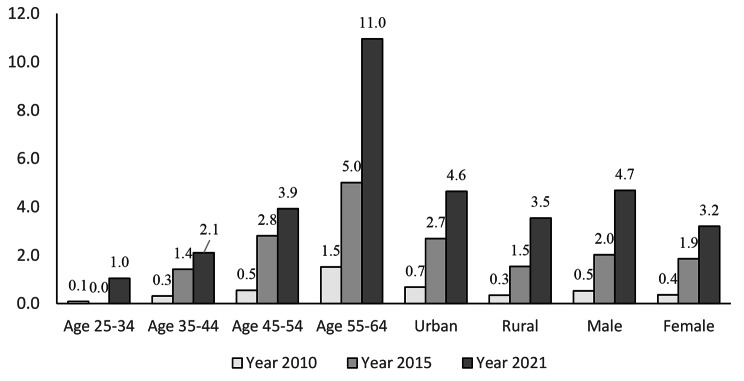
Trends of T2DM-HTN comorbidity by sub-population groups 2010–2021

Figure 2 illustrates the trends in T2DM-HTN comorbidity during the last 10 years across different sub-population groups. All sub-populations presented consistently increasing trends over time, with the sharpest increase among the 55–69 years old age. Regarding absolute change, within the oldest age group, the T2DM-HTN percentage surged from 1.5% in 2010 to 5.0% in 2015 and further to 11.0% in 2021. When considering relative change, the burden of T2DM-HTN comorbidity in this age group escalated approximately 7.3 times over the past decade.

This upward trend of T2DM-HTN percentage was evident in both rural and urban populations. While urban areas experienced a larger absolute change over the course of a decade (an increase of 3.9% compared to 3.2% in rural areas), the relative change was more significant in rural settings, where it increased by 11.3 times, as opposed to 6.7 times in urban areas. In addition, the male population displayed a higher trend in comorbidity compared to the female population, both in relative and absolute changes.

### Factors associated with comorbidity of diabetes and hypertension

The multivariate logistics model (Table 3) confirmed that adjusted for the difference in characteristics of the population samples over time (i.e., age differences, geographical area, gender, BMI, and total NCD behavioral risk factors), the increasing trends of T2DM-HTN comorbidity remained statistically significant. Compared to 2010, the adjusted ORs of T2DM-HTN comorbidity for the year 2015 and 2021 was 3.58 and 7.4 (*p* < 0.001), respectively.

The adjusted ORs of the female population compared to the male and adjusted ORs of the rural population compared to the urban population was < 1.0, indicating lower risks of T2DM-HTN comorbidity among these subgroups.

The positive association between BMI score and T2DM-HTN comorbidity was indicated in the model; people with a BMI equal to or greater than 25 would have a higher risk of the study outcomes (adjusted ORs = 2.91, *p* < 0.001). The adjusted ORs of T2DM-HTN comorbidity increased incrementally as the number of NCD behavioral risk factors increased. However, only people with all NCD behavioral risk factors (i.e., current smoking, current drinker, not meeting WHO recommendation for physical activity, not consuming enough 5 servings of fruit/vegetable daily) would have a statistically significant association with the outcome (adjusted ORs = 3.48, *p* < 0.001).

**Table 3:** Final multivariate logistic model for T2DM-HTN comorbidity

*Factors*	*Adjusted ORs*	*95% CI ORs*	*p-value*
***Year 2010***	***1.00***		
Year 2015	3.58	[2.54, 5.05]	< 0.00
Year 2021	7.40	[5.63, 9.72]	< 0.00
***Male***	***1.00***		
Female	0.90	[0.68, 1.19]	0.47
***Urban***	***1.00***		
Rural	0.75	[0.59, 0.96]	0.02
***Age 25–34***	***1.00***		
Age 35–44	2.91	[1.34, 6.32]	0.01
Age 45–54	6.95	[3.35, 14.38]	< 0.00
Age 55–65	14.12	[6.92, 28.81]	< 0.00
High BMI (BMI≥25)	2.91	[2.28, 3.73]	< 0.00
None of the risk factor*	***1.00***		
Have one risk factor*	1.02	[0.71, 1.47]	0.91
Have two risk factors*	1.05	[0.71, 1.57]	0.79
Have three risk factors*	1.18	[0.73, 1.92]	0.50
Have all four risk factors*	3.48	[1.92, 6.33]	< 0.00

*Four risk factors: current smoking, current drinker, not meeting WHO recommendation for physical activity, not consuming enough 5 servings of fruit/vegetable daily.

## Discussion

NCD prevention and control has been one of Vietnam’s health priorities [14]. This study combined the data from the three STEPs surveys to estimate the current burden of T2DM-HTN comorbidity, the trends over time, and factors correlated with comorbidity. Results showed that in 2021, about 3.92% of Vietnam’s population aged 25–64 years old had both diabetes and hypertension. This figure was higher among men compared to women (4.7% vs. 3.2%). A previous study utilized the Bangladesh Demographic and Health Survey (BDHS) dataset from 2017 to 2018 and reported a prevalence of 4.47% for T2DM-HTN comorbidity [15]. In another study conducted in India using STEPs data from 2017, the reported prevalence was 4.5% [16]. A study in Malaysia reported that 57.9% of people with diabetes also had hypertension but this study did not report the prevalence of comorbidity among the general population [17]. However, determining whether the prevalence of comorbidity among the Vietnamese population was high or low compared to other Asian countries should take into account potential variations in sample sizes, age structures, and survey methodologies across studies.

This study also revealed a significant increasing trend over time for T2DM-HTN comorbidity. During the last 10 years, the burden of T2DM-HTN comorbidity among people aged 25–64 in Vietnam has increased more than 8 times (from 0.44 to 3.92%). The upward trends of T2DM-HTN comorbidity were repeatedly observed for all sub-population analyses. The most dramatically increasing trends happened among the old age group (i.e., age 55–64) and the male population. The multivariate logistics model confirmed the statistical significance of the trends after adjusting for the changes in socio-demographic factors, NCDs, and behavioral and metabolic factors of population samples over time. A previous study using the same data sources presented detailed information about the increasing trends of hypertension and diabetes [4]. The precise cause of the rising trend in comorbidity between diabetes and hypertension remains unclear. Previous studies reported potential explanations for this rising trend including lifestyle changes, such as unhealthy diets, sedentary lifestyles, insufficient physical activity, environmental influences, and the aging process [18].

The increasing comorbidity burden between hypertension and diabetes represents a significant health challenge due to their shared risk factors, biological mechanisms, and potential complications. People with T2DM-HTN comorbidity have a higher risk of macrovascular complications, including stroke or coronary heart disease (12). Hypertension also facilitates the progression and complication of diabetic nephropathy and retinopathy [11]. Controlling blood pressure and blood sugar in T2DM-HTN patients will be more difficult than in patients with only one of the two diseases [19].

Like other low and middle-income countries, Vietnam started to regulate NCD management in primary health care, especially at commune health stations (CHSs) in 2018 [20]. Within Community Health Stations (CHSs) in Vietnam, patients with both hypertension and diabetes are presently being managed through separate vertical lists. However, the substantial prevalence of comorbidity is striking, with an average of 13.8 patients per 100 hypertension cases and 56.3 patients per 100 diabetes cases presenting comorbidity. This underscores the pressing requirement for integrated management initiatives, particularly at the CHS level, to address the needs of individuals suffering from both hypertension and diabetes.

### Top of form

Prior research frequently linked shared risk factors—such as high BMI, insufficient physical activity, and elevated cholesterol levels—as contributors to the comorbidity of T2DM-HTN [10, 21]. However, only a few studies ever explicitly examined the impacts of these factors on comorbidity. One study about hypertension among the Diabetes Population in Malaysia in the year 2015 reported that age group, working status, obesity, and cholesterol were associated with hypertension among respondents with diabetes [17]. Another study used data from the 2008–2011 Korea National Health and Nutrition Examination Surveys and concluded that body fat variables were significant predictors for the comorbidity of T2DM-HTN [22]. In this study, BMI showed a significant association with the coexisting condition of these two diseases. The odds of having comorbidities were 1.19 times higher when BMI increased by 1 kg/m^2^. This result confirmed the previous observation that increased BMI resulted in a higher risk for type 2 diabetes and hypertension and that most patients with diabetes/hypertension have a BMI≥25 [23] as well as the finding from the Korean study [22]. This study took the recommendations of prior research [24, 25] on investigating the clustering effects of NCD behavioral risk factors. A clustering behavioral risk score was devised, including 4 factors (i.e., smoking, alcohol consumption, failure to meet WHO recommendations for physical activity, and inadequate daily consumption of five servings of fruits and vegetables). The findings revealed a positive correlation between the number of risk factors present and the likelihood of comorbidity between diabetes and hypertension. Specifically, the adjusted ORs for comorbidity were as follows: 1.02 for individuals with 1 risk factor, 1.05 for those with 2 risk factors, 1.18 for those with 3 risk factors, and 3.48 for individuals with all 4 risk factors. Notably, statistical significance was observed solely in the ORs about the group exhibiting all 4 risk factors (OR=3.48) compared to those without any risk factor. Smoking, alcohol consumption, not enough physical activities, and less daily fruit/vegetable consumption have been reported to be significant risk factors for many NCDs, including hypertension and diabetes [26–29]. Previous studies mostly examined the effects of these behavioral risk factors on NCDs separately, few have explored the clustering effects of these factors combined [24, 25]. The STEPs survey used standardized questionnaires based on the WHO’s STEPS approach [12] for data collection. The survey sample sizes for all rounds were designed to be a nationally representative survey and provide valid estimates of NCD risk factors among the general population and stratification by age, gender, and rural/urban. The combination of STEPs data from the three surveys in Vietnam gave this study valid and nationally representative data sources to examine the trends of hypertension, diabetes, and their comorbidity over the last 10 years in Vietnam and some factors contributing to the comorbidity status. However, due to the absence of specific information provided in the original survey data, the study could not identify factors fully responsible for these observed trends. Further, longitudinal studies are needed to identify changes in population over time, accounting for the changes in the burden of T2DM-HTN comorbidity.

The significant association between age, gender, rural/urban, BMI, and clustering NCD behavioral risk factors in this study can be used as evidence to identify groups with a higher burden of comorbidities in the population for future intervention.

## Conclusion

The current burden of T2DM-HTN among the population aged 25–64 years old in Vietnam is high. The significant increasing trends over the last 10 years of T2DM-HTN comorbidity were observed for all populations aged 25–64 and for all sub-population analyses. Sub-populations demonstrating the most significant changes included the male population, people living in urban areas, and older people.

The high comorbidity between hypertension and diabetes underscores the imperative for integrated treatment and management approaches in Vietnam. Coordinated care is essential for addressing the complex interplay between these two prevalent conditions.

## Data Availability

The datasets used and/or analyzed during the current study are available from the corresponding author upon reasonable request.
